# Unveiling the complexity of transcription factor networks in hematopoietic stem cells: implications for cell therapy and hematological malignancies

**DOI:** 10.3389/fonc.2023.1151343

**Published:** 2023-06-27

**Authors:** Aissa Benyoucef, Jody J. Haigh, Marjorie Brand

**Affiliations:** ^1^ Department of Pharmacology and Therapeutics, Rady Faulty of Health Sciences, University of Manitoba, Winnipeg, MB, Canada; ^2^ CancerCare Manitoba Research Institute, Winnipeg, MB, Canada; ^3^ Sprott Center for Stem Cell Research, Ottawa Hospital Research Institute, Ottawa, ON, Canada; ^4^ Department of Cellular and Molecular Medicine, University of Ottawa, Ottawa, ON, Canada

**Keywords:** hematopoietic stem cells, transcription factor, reprogramming, cell therapy and hematological malignancies, trans-differentiation

## Abstract

The functionality and longevity of hematopoietic tissue is ensured by a tightly controlled balance between self-renewal, quiescence, and differentiation of hematopoietic stem cells (HSCs) into the many different blood lineages. Cell fate determination in HSCs is influenced by signals from extrinsic factors (e.g., cytokines, irradiation, reactive oxygen species, O2 concentration) that are translated and integrated by intrinsic factors such as **Transcription Factors** (TFs) to establish specific gene regulatory programs. TFs also play a central role in the establishment and/or maintenance of hematological malignancies, highlighting the need to understand their functions in multiple contexts. TFs bind to specific DNA sequences and interact with each other to form transcriptional complexes that directly or indirectly control the expression of multiple genes. Over the past decades, significant research efforts have unraveled molecular programs that control HSC function. This, in turn, led to the identification of more than 50 TF proteins that influence HSC fate. However, much remains to be learned about how these proteins interact to form molecular networks in combination with cofactors (e.g. epigenetics factors) and how they control differentiation, expansion, and maintenance of cellular identity. Understanding these processes is critical for future applications particularly in the field of cell therapy, as this would allow for manipulation of cell fate and induction of expansion, differentiation, or reprogramming of HSCs using specific cocktails of TFs. Here, we review recent findings that have unraveled the complexity of molecular networks controlled by TFs in HSCs and point towards possible applications to obtain functional HSCs *ex vivo* for therapeutic purposes including hematological malignancies. Furthermore, we discuss the challenges and prospects for the derivation and expansion of functional adult HSCs in the near future.

## 1 Introduction

Hematopoietic tissue is composed of different cell types (e.g., erythroid, lymphoid). These cells have different lifespans depending on the physiological state of each individual and must be produced continuously through life. This sustainability is maintained by self-renewal/proliferation and differentiation of a small contingent of cells called hematopoietic stem cells (HSCs), which are at the top of the hematopoietic hierarchy. The identification and characterization of HSCs began in the early 1960s with the seminal work of Till and McCulloch (1). This was followed by a number of studies culminating in the development of methods to purify enriched populations of these cells using various parameters such as size, granularity and specific combinations of cell surface markers (2).

**Figure 1:**
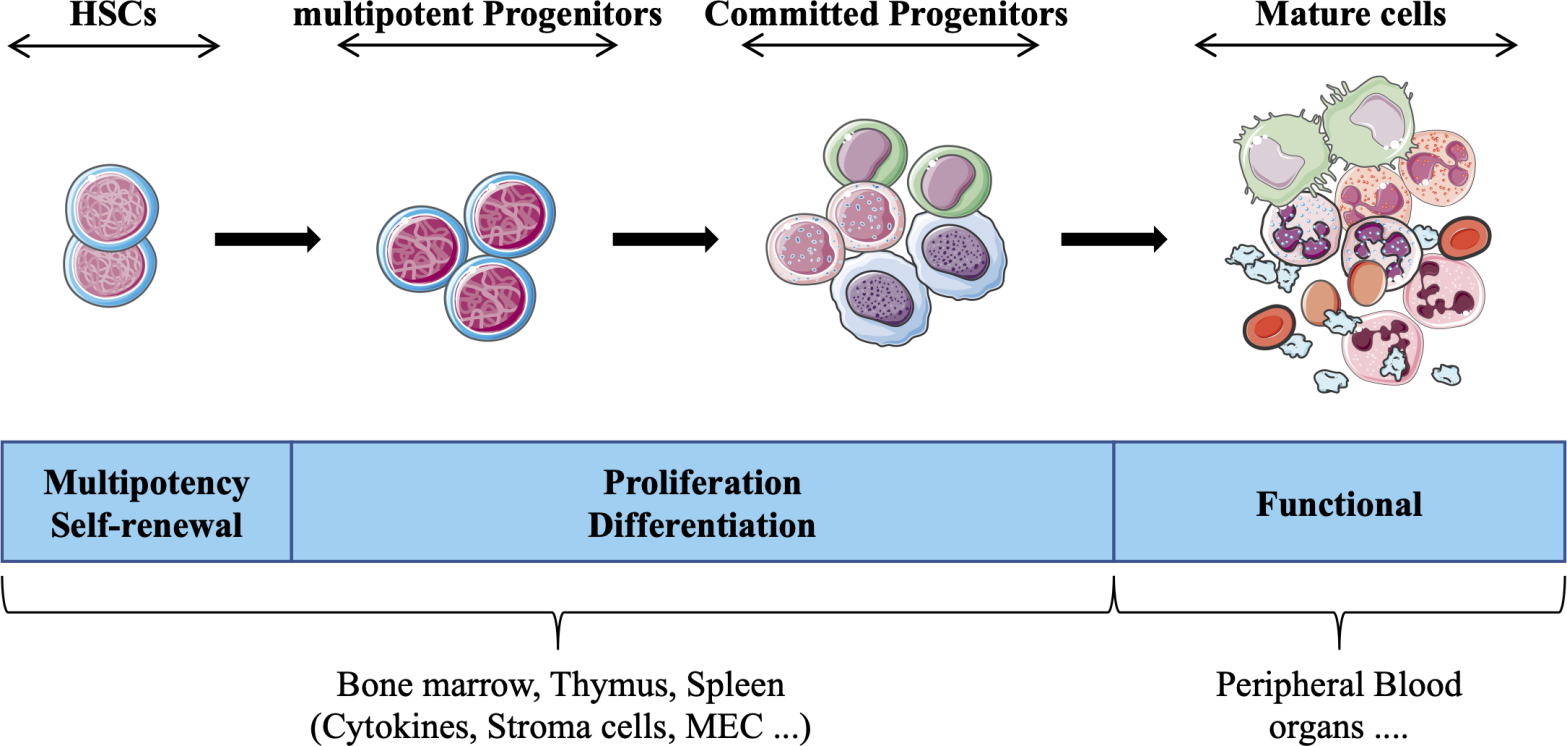
Schematic representation of hematopoietic tissue according to the currently prevailing working model, adapted from (3). Hematopoietic stem cells (HSCs) give rise to daughter cells under various physiological conditions (e.g., cytokines) daughter cells, i.e. multipotent and committed progenitor cells that migrate to different hematopoietic organs (e.g. thymus), which in turn form the basis for all mature blood cell types that provide the different functions of the hematopoietic tissue.

HSCs exist in a specific microenvironment provided by the bone marrow, which together with extrinsic factors (e.g., cytokines, reactive oxygen species, O2 concentration) contribute to determining the fate of these cells. The interaction of hematopoietic stem/progenitor cells with different microenvironments (e.g., spleen, thymus) strongly influences differentiation and proliferation, such that cells acquire specific functional and phenotypic characteristics that define each hematopoietic lineage (e.g., lymphoid, myeloid). Historically, cells that constitute the hematopoietic system have been divided into four compartments (HSCs, multipotent progenitors, committed progenitors and mature cells) that are interconnected in a complex hierarchical system (**Figure 1**). Each compartment is heterogeneous and forms a continuum of cells representing a stage of maturation and differentiation. While this model is useful for understanding the regulation of hematopoiesis, alternative hierarchical organizations have also been proposed (4, 5). Furthermore, technological advances including the use of single-cell assays, have unveiled heterogeneity of the HSC compartment (6, 7), and other studies have suggested that HSCs display various degree of differentiation biases (e.g., lymphoid (8), myeloid (9), or megakaryocyte/platelets (10) lineages). These findings have challenged the traditional model of a hierarchical hematopoietic system and led to an alternative model, wherein the acquisition of lineage-specific fates is a continuous process (11).

Extrinsic factors influence hematopoiesis through the activation or suppression of signaling pathways, including BMP and WNT that are important for the state of HSCs such as quiescence, self-renewal and/or differentiation. These signaling pathways function through a complex mechanism of activation and/or repression of transcription factors (TFs), which in turn directly or indirectly regulate specific groups of genes necessary to promote or inhibit differentiation (12).

Here we will review the importance and complexity of TF-based mechanisms regulating hematopoiesis, with a particular focus on HSCs. We will also highlight specific examples of how TFs can be manipulated to derive functional HSCs, which may represent key therapeutic strategies for various hematological diseases in the future.

## 2 Transcription factors

TFs are defined as proteins that bind to DNA in a sequence-specific manner to regulate transcription. TFs have the ability to interact with each other and form stable protein complexes bound to cis-regulatory elements (CREs). By definition, CREs are short genetic elements (typically shorter than 1 Kb) that act as regulatory switches to control gene expression. CREs are divided into gene promoters or distal elements called enhancers/silencers (> 1,000 bp away from a transcription start site (TSS)), and insulators that form regulatory boundaries between genomic domains. Interestingly, the distal elements or enhancers/silencers are most important for activating regulatory programs that control cellular identity (through self-renewal) and differentiation of HSCs into different cell lineages (13).

The availability and localization of TFs in the nucleus, their relative affinity for each other’s and for specific motifs in CREs determines the complexity and composition of TF complexes and their ability to recruit cofactors, including the epigenetic regulatory machinery (coactivators, co-repressors, and chromatin remodeling proteins). Cofactors, in turn, act to activate or repress transcription of specific sets of genes through various mechanisms. These include recruitment and activation/repression of the machinery associated with RNA polymerase II (e.g., NELF, Mediator)(14, 15), alterations of the chromatin landscape, and three-dimensional (3D) nuclear positioning of loci relative to transcription factories (16–19) or to the phase-separated multi-molecular complex of transcriptional regulators (20) within the nucleus.

TFs can be classified according to their 3D structure and protein sequence similarity of their DNA-binding domain (e.g., zinc finger, helix-turn-helix, helix-loop-helix, basic leucine zipper, homeodomain, winged helix). TF families are well conserved throughout evolution (21) and in various biological systems (e.g., nervous, muscle, and hematopoietic systems) (22). However, some TFs are restricted to specific lineages. For example, within the hematopoietic lineage, PAX5 is expressed exclusively in B lymphoid cells (23), whereas GATA1 is expressed at higher levels in mega/erythroid cells (24). Interestingly, structural similarities of TFs from the same family can lead to functional redundancy. For example, Scl/Tal1 and Lyl1 encode two very similar bHLH TFs. In adult mouse HSCs, deletion of these TFs separately has no effect on HSC survival and potential, but deletion of both TFs in HSCs leads to massive apoptosis and impaired function of hematopoietic lineages (25).

## 3 Investigating the role of TF levels in the regulation of hematopoiesis and HSCs

Several studies using transgenic mice (26) and viral vectors (e.g. retrovirus (27), lentivirus (28)) to knockdown or overexpress specific TFs in a tissue-specific manner have helped to establish the basic principles defining the function of TFs in hematopoietic tissues and HSCs. In particular, the balance between quiescence, self-renewal, and differentiation of HSCs was found to be finely regulated by specific groups of TFs, some of which are required during quiescence, while others regulate self-renewal and differentiation. Importantly, disruption of TFs can lead to major diseases, including (**1**) aplastic anemia resulting from premature depletion of the HSC pool due to excess differentiation or massive apoptosis; (**2**) leukemia resulting from inhibition of differentiation and uncontrolled proliferation; (**3**) permanent quiescence resulting from blockade of the cell cycle, self-renewal, and differentiation.

Complementary to the functional studies described above, technical advances in cell purification techniques using flow cytometry have led to greater refinement of our knowledge of the different populations that make up hematopoietic tissue (5, 29, 30), while high-throughput genomic (e.g., ChIP sequencing, RNA sequencing) and proteomic approaches led to a better understanding of TFs at the molecular level and enabled the discovery of novel TFs and cofactors involved in the regulation of hematopoiesis (31).

## 4 Context-dependent functions of TFs in the regulation of hematopoiesis

While hematopoietic TFs are often analyzed separately, some studies have examined how TFs work together and by what mechanisms they respond to environmental signals (12). For example, integrative analysis of 38 hematopoietic TFs in different cell populations sorted from cord blood using specific cell surface markers revealed that cells at different stages of hematopoietic differentiation express specific combinations of TFs (32). Furthermore, HSCs express high levels of TFs required to maintain the undifferentiated/quiescent state (e.g., MEIS1, ERG) (33, 34) and TFs that actively suppress differentiation (e.g., GFI1, GFI1b) (35, 36). For instance, studies using mouse models have demonstrated the role of several TFs (e.g., GATA2, TAL1, RUNX1) in the formation of both primitive and definitive hematopoietic stem cells (HSCs) during embryonic development (previously reviewed (37, 38)). Indeed, knockout of *Gata2* at the embryonic stage showed is essential for the HSC formation from hemogenic endothelium, a process termed endothelial to hematopoietic transition (EHT) (39–41). In adult mice, deletion of *Gata2* leads to apoptosis and impaired proliferation of HSCs. However, deletion of a single allele of *Gata2* reduces the HSC pool without affecting their viability (42). Conversely, overexpression of GATA2 in murine HSCs using a retrovirus strategy decreases their ability to generate colony-forming cells (CFCs) *in vitro* and impairs their capacity to restore hematopoiesis in irradiated mice. Interestingly, ectopic expression of GATA2 does not affect the cells’ ability to engraft upon transplant, but it does reduce their potential to proliferate and differentiate *in vivo* (43). Also interestingly, changes in GATA2 levels through precise manipulation of enhancers in hematopoietic progenitors *in vivo* revealed the critical role of GATA2 in cell fate determination (44). In human HSCs isolated from cord blood, ectopic expression of GATA2 increases their quiescence without inducing apoptosis. It does not impact their ability to transplant in immunodeficient mice (NOD-SCID) but reduces their potential to differentiate *in vivo*, depending on the degree of GATA2 overexpression, even in secondary transplantation (28). In summary, the results of these studies highlight the context-dependent function of GATA2 in embryonic and adult HSCs. Additionally, they underscore the importance of GATA2 expression level in the maintenance of HSCs in an undifferentiated and quiescent state, potentially preventing their depletion over time.

Also, perturbation of this equilibrium by extrinsic signals (e.g., growth factors produced by the environment) through disruption of the interactions of TFs is necessary to trigger differentiation into a particular cell lineage. For example, suppression of WNT signaling in HSCs has been shown to modulate the activity of TCF TFs (45–47), resulting in decreased expression of genes involved in HSC maintenance and increased expression of TFs that promote erythroid differentiation (e.g., PBX1 and GATA1) (24, 32, 48).

Another important aspect of differentiation mediated by TFs is the fact that certain TFs that promote differentiation to a particular hematopoietic lineage are usually also actively involved in the repression of genes from alternative lineages. An example of this is PU.1, a transcription factor that promotes granulo/macrophage differentiation and B-cell lymphopoiesis while actively repressing genes of T cell lymphopoiesis such as pre-Tα, Rag-1, and Rag-2 when ectopically expressed in T lymphocytes (49).

## 5 Context-dependent expression of TFs in hematopoiesis

**Figure 2:**
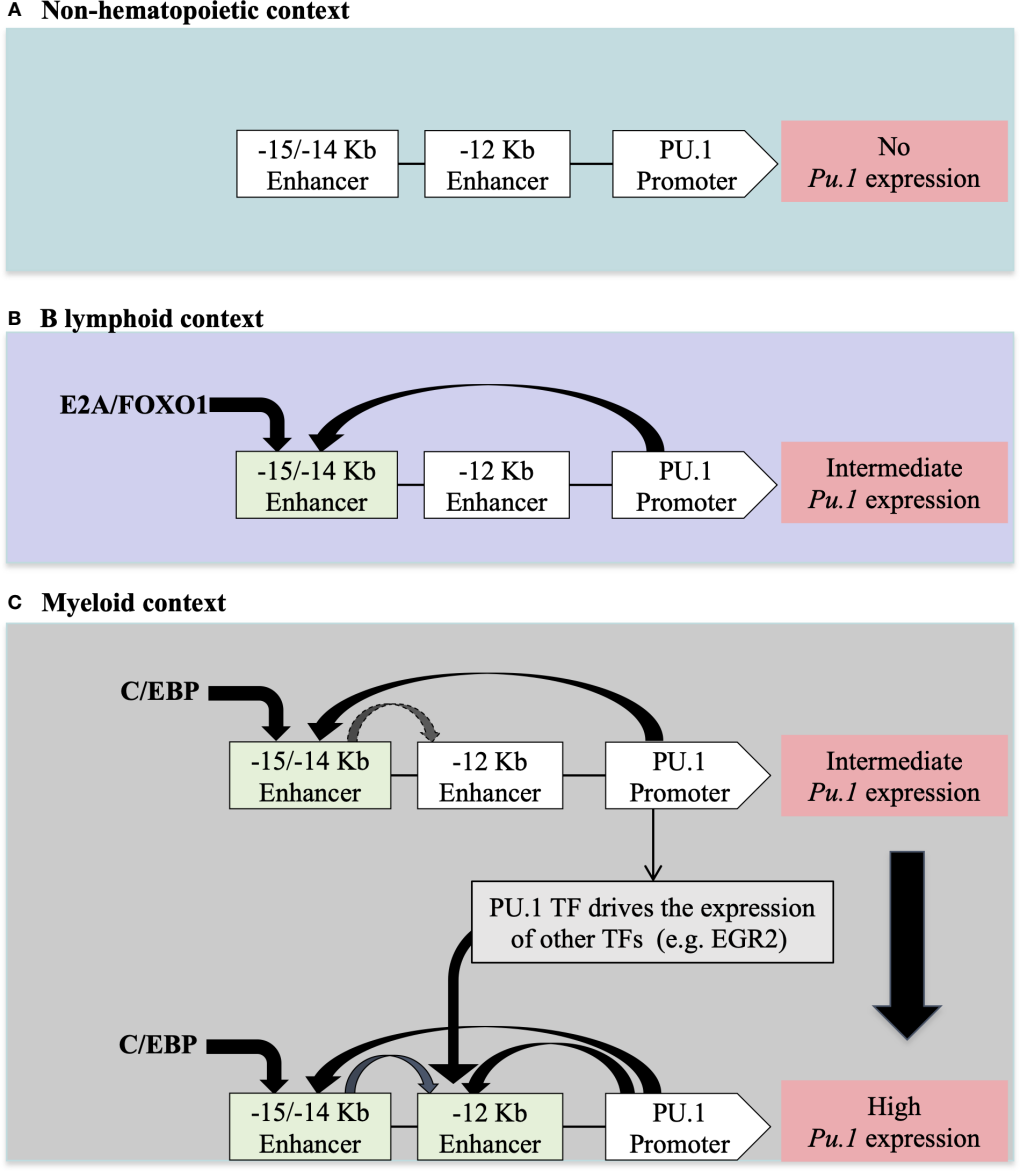
The regulation of *PU.1* locus expression in different cellular contexts according to (50) **(A)** In non-hematopoietic cells, the absence of *PU.1* expression is due to the absence of hematopoietic TFs that regulate expression in hematopoietic tissue. **(B)** The presence of E2A/FOXO1 TFs in lymphoid B cells allows for intermediate expression of PU.1 TF through a -15/-14 Kb enhancer **(C)** The presence of C/EBP/EGR2 TFs in myeloid cells allows for high-level expression of *PU.1* through -15/-14 Kb and -12Kb enhancers upstream of the *PU.1* promoter.

Many TFs are expressed in different hematopoietic lineages. However, their expression levels may differ depending on the cell type. For example, PU.1 is differentially expressed in myeloid versus lymphoid cells. This cell type-specific expression level is controlled by other TFs that recognize CREs upstream of the *Pu.1* promoter. For example, in myeloid cells, C/EBP binds to two CRE regions located at -15Kb/-14Kb and at -12Kb upstream of the *Pu.1* promoter to ensure strong expression of PU.1 in this context. In contrast, in B lymphoid cells, where *Pu.1* expression is lower, the E2A and FOXO1 TFs only bind to the -15Kb/-14Kb CRE region upstream of the *Pu.1* promoter (50) (**Figure 2**).

This tightly controlled expression of TFs in hematopoietic tissues is also mediated by distinct CREs that differentially regulate the expression of the same TF in specific cells allowing for fine-tuned TF expression. For example, deletion of a 2.4 Kb region located +19 Kb downstream of the *Tal1* promoter decreases its expression specifically in HSCs (51). However, deletion of a 1.3 Kb region located at +40 kb downstream of the *Tal1* promoter decreases its expression specifically in the erythroid lineage without affecting its expression in other lineages (52). Interestingly, leukemic T cells (in which TAL1 is a potent oncogene) also exhibit a specific CRE region that drives *TAL1* expression in the T cell lineage. This *de novo* CRE region arises from the abnormal insertion of nucleotides at -7Kb upstream of the *TAL1* promoter, which enables the abnormal activation of TAL1 in the T cell lineage (53, 54).

While cell-specific CREs regulate the transcription of genes encoding TFs, it is important to note that transcript abundance does not always correlate with protein levels (55) and that TF genes are subject to a number of post-transcriptional regulatory mechanisms. For example, the microRNA MiR-150 targets the transcript of c-MYB TF in immature hematopoietic progenitor cells to promote B-cell differentiation (56). Regulation can also occur at the translational level. For example, the expression of TAL1 TF isoforms in different hematopoietic lineages (e.g., HSCs, erythrocytes, megakaryocytes) is regulated by distinct signaling pathways that modulate translation initiation factor functions in each lineage (57). As for the regulation of TFs at the protein level, this can be modulated by changes in the microenvironment, such as the increase of oxygen concentration in the perivascular niche, which is known to enhance the degradation of HIFα-TFs in HSCs (58, 59), or by the activation of signaling pathways such as the SMAD/TGFβ pathway, which phosphorylates TAL1 at threonine 90 and at serine 122 to trigger its degradation under hypoxic conditions (60, 61). On the other hand, phosphorylation of TAL1 at serine 172 destabilizes its interaction with the epigenetic cofactor LSD1 and subsequently increases the expression of its target genes by inducing H3K4 hypermethylation at the promoters of TAL1 target genes (62). Moreover, acetylation of TAL1 at lysine 221/222 increases its DNA-binding ability and transcriptional activity (63).

In summary, the expression of TFs in hematopoietic tissues is spatially and temporally regulated at the transcriptional, posttranscriptional, translational, and posttranslational levels. Their specific expression in a given cell context is regulated by (1) a set of TFs already expressed in each specific cellular environment (e.g., HSCs), (2) by various extrinsic factors (e.g., hypoxia) (3), and also by certain intrinsic factors (e.g., microRNA). The newly expressed TFs, in turn, interact with other TFs to recognize specific CRE regions to ensure appropriate expression levels of their target genes.

## 6 Investigation of TF networks in HSCs

**Figure 3:**
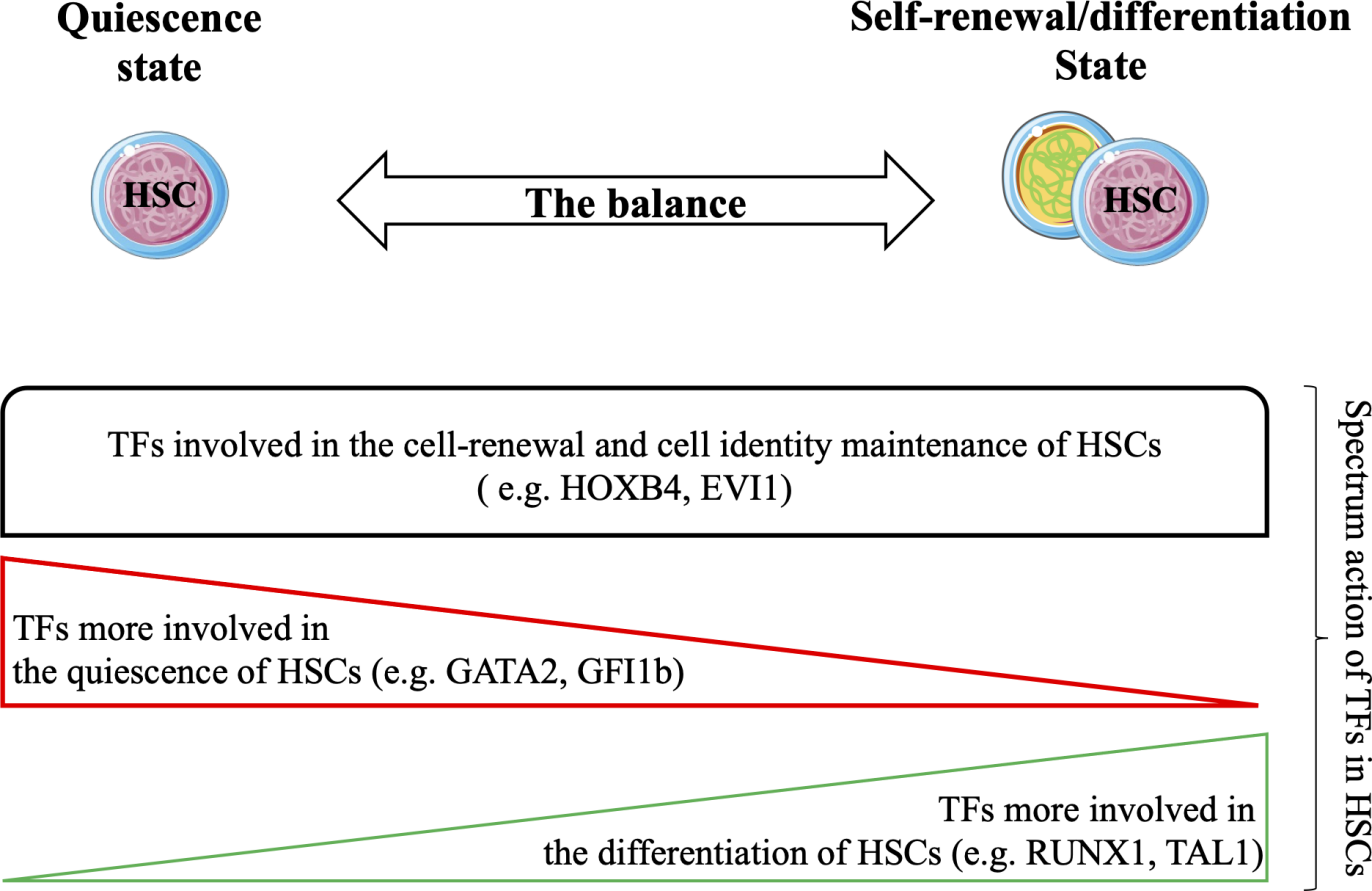
Hematopoietic stem cells (HSCs) balance between the quiescent state and the cyclic state (for self-renewal/differentiation) to maintain the durability of hematopoietic tissue during the lifespan. Several studies have confirmed the strong involvement of TFs in all states of HSCs. Functional experiments have shown that TFs are involved in cell renewal and maintenance of cell identity of HSCs (e.g. HOXB4, EVI1). Certain TFs are required for their quiescence (e.g. GATA2, GFI1) and others are more required during differentiation of HSCs (e.g. RUNX1, TAL1). Taken together, these TFs may form transcriptional networks that control the functions of HSCs.

HSCs are finely regulated to maintain hematopoietic tissue homeostasis. HSCs are mainly in a quiescent state, and under various stimuli, they enter the cell cycle to self-renew and/or differentiate. The process of HSCs transitioning from the quiescent state to the cyclic state for self-renewal or differentiation is regulated by a specific set of TFs (**Figure 3**) that play synergistic or antagonistic roles in the HSC compartment. Together, these TFs form distinct networks that control HSC functions. Recent studies have attempted to unravel TF networks by using various high-throughput strategies that explore their complexity and interactions.

In the next section, we review recent advances in our understanding of the role of TF networks in regulating the function of adult HSCs. Understanding the molecular networks that govern HSC biology is expected to improve therapeutic applications involving adult HSCs (e.g., cell therapy (64)) and will also pave the way for the development of other new innovative therapies (e.g., immunotherapy (65) and mutation/gene-correction in HSCs with the CRISPR system (66)).

### 6.1 Using cis-regulatory elements to explore TF networks in HSCs

Self-renewal and multipotency are key properties of HSCs that require the timed activation of specific gene groups. This process is largely controlled by the binding of TFs to CREs (67). The first generation of TF networks was created mainly using CRE regions known to be associated with genes expressed in HSCs. Indeed, the expression level of TFs obtained from the microarray data of several studies was analyzed using mathematical models to predict the set of TFs interacting with CRE regions in HSCs. For example, by using the mathematical models to study the CRE regions in the HSC context, it was shown that the TAL1-GATA2-FlI1 network is essential for HSC development and its activity can be modulated by physiologically relevant signals such as Notch, Bmp4, and GATA1(68). In another study, it was shown that this TF network may be more complex, containing additional TFs (e.g., RUNX1, HHEX, and ERG), and that GATA1 can disrupt this TF network by simply repressing Fli1 expression to trigger differentiation of HSCs (32, 68–72).

Recently, new high-throughput methods have been developed to map all CRE regions in HSCs and in other cell types. In principle, the active CRE corresponds to accessible chromatin where nucleosomes are less tightly packed and is hypersensitive to digestion by DNase I. By combining this property with high-throughput sequencing, it is possible to use this method (DNaseI-seq) to generate a genome-wide profile of the landscape of the open chromatin (CREs) of any cell type. To address the problem of large amounts of starting material for rare populations such as HSCs, another method has been developed using a bacterial transposase (Tn5). This Tn5 enzyme is used to insert two short DNA fragments (a transposon) on either side of accessible chromatin regions. This assay for transposase-accessible chromatin with high-throughput sequencing (ATAC-seq) is comparable to DNaseI-seq in terms of specificity and signal-to-noise ratio, achieving 100- to 10000-fold with fewer cells than DNaseI-seq (73). Application of ATAC-seq in different hematopoietic cell types has shown that HSCs have a higher number of CREs than all other hematopoietic progenitor cells (megakaryocytes, erythrocytes, granulo-macrophages, B and T lymphocyte progenitor cells). Interestingly, megakaryocyte progenitors (MkPs) are the unipotent cell type most closely related to HSCs because they lose fewer “HSC” CREs. However, B lymphocyte progenitors (BPs), like MkPs progenitors, lose fewer “HSC” CREs but acquire more “*de novo*” CREs. Thus, CREs in HSCs can be divided into two categories: the specific CREs, which are active only in the HSC, and the “primed” CREs, which are present in different progeny of unilineage progenitors and could be responsible for binding TFs without being transcriptionally active (74). Motif analysis of CREs in the context of HSCs compared to other unilineage progeny confirmed the cell type-specific enrichment of transcription factor motifs. For example, the binding motif PAX is strongly represented in CREs from BPs in which PAX5 plays an important role. On the other hand, the binding motif GATA is highly enriched in CREs of megakaryocytic-erythroid progenitors in which GATA1 plays an important role. However, in the context of HSCs, there is enrichment for several TFs such as NFIC, STAT3/4 and HOX TFs (13). Moreover, restricted motif enrichment analysis of CREs present only in HSC compartments (LT-HSCs, ST-HSCs and MPP) reveals specific motif enrichment for Kruppel-like TFs (KLF9, KLF10 and KLF14) and ETV2 TF in LT -HSCs (75).

Profiling of proximal CREs (at promoter level) associated with genes expressed in HSCs reveals that certain TFs (more than 20 TFs) are tightly connected by autoregulation, feedback and autoactivation (wherein a factor directly or indirectly activates its own expression). These mechanisms work together to establish a resilient transcriptional network capable of sustaining the expression of genes essential for HSC biology. During differentiation, the network of TFs expressed by HSCs gradually disappears as the corresponding TFs are no longer expressed along several lineages. At the same time, other dense TF networks emerge through the induction of other TFs associated with terminally differentiated cells, such as GATA1, LMO2, FOXO4, NFE2, and RXRA, which are involved in the TF network associated with erythroid differentiation. Certain TFs involved in TF networks associated with the functions of HSCs remain expressed and are “re-used” throughout the unilineage progeny. For example, PBX1 is involved in a TF network that regulates self-renewal of HSCs, but may later interact with other TFs to establish a TF network required for erythroid and megakaryocyte progenitor cell differentiation (32).

Interestingly, the robustness of this strategy for predicting the TF networks controlling the functions of HSCs was tested using a perturbation-based approach. This led to validation of 17 predicted TFs involved in TF networks generated during differentiation of HSCs into different lineages (erythroid or myeloid). Specifically, by using conditioned medium that supports both erythroid and myelo-monocytic differentiation, the differentiation of enriched HSC cells into either of these lineages could be altered by simply reducing the expression of one of the predicted TFs. For example, altering the expression of 9 TFs (such as GATA1 and KLF1) affects the differentiation of HSCs toward the erythroid lineage without affecting their potential to differentiate toward the myeloid lineage. Moreover, using the CREs strategy to predict the TF network associated with the erythroid lineage reveals three novel TFs not previously associated with erythroid differentiation (AFF1, HIF3A, and YY1 TFs). In contrast, disruption of the expression of the 7 TFs (such as PU.1/SPI1 and members of the C/EBP family) involved in the TF network that drives differentiation of HSCs toward the myeloid lineage does not affect their differentiation toward the erythroid lineage but drastically reduces their potential for myelomonocytic differentiation (32).

### 6.2 Using a protein-based approach to explore TF networks in HSCs

The approach of using motif enrichment analysis of CRE regions in combination with the transcriptional level of TFs to establish TF networks in HSCs may have some limitations. Firstly, certain genes are regulated by multiple CREs that can be specifically used in different cell contexts to maintain their expression. Thus, all CREs identified in HSCs should in principle be validated experimentally to confirm their specificity and relevance to the HSC context by using transgenic mice or CRISPR/Cas system for human cells. For example, the *Erg* locus contains five CREs regions (+65Kb, +75Kb, +85Kb, +90Kb, and +149Kb upstream of the *Erg* locus), of which only the +85Kb region has been validated in transgenic mice (76). However, the other four Erg CREs have not yet been validated and their integration into a CRE analysis may not be necessary or relevant to build the TF network in the HSC context. Secondly, the mRNA level may not correlate with the protein level of TFs in hematopoietic tissue. For example, BCL11A, a master regulator of fetal hemoglobin remodeling, is regulated at the level of mRNA translation by the RNA-binding protein LIN28B during hematopoietic development (77). Therefore, post-transcriptional regulatory mechanisms may vary between different cellular contexts (78), and using only the transcriptional level of TFs to construct TF networks in HSCs may lead to some imprecision. The two main concerns mentioned previously have led to the emergence of antibody-based approaches to study the biology of TFs in hematopoietic tissues. The rapid decline in sequencing costs has accelerated the generation of genome-wide TF binding maps by coupling chromatin immunoprecipitation with high-throughput sequencing (ChIP-Seq) (79).

**Figure 4:**
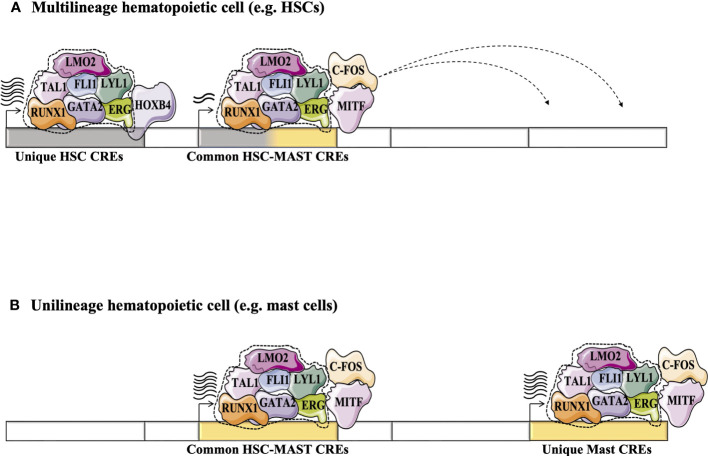
Exploring the dynamics of TFs in HSCs and during their differentiation (e.g., in differentiated mast cells) using the high-throughput sequencing strategy adapted from (82). **(A)** Statistical analysis of 10 TFs-ChIP-seq has revealed a novel TF network consisting essentially of the heptad factors (SCL, LYL1, GATA2, LMO2, ERG, FLI1 and RUNX1) that plays an important role in regulating genes specifically expressed in HSCs (81). **(B)** In mast cell progeny, the 7 TFs network integrates other TFs such as MITF and c-FOS to regulate the expression of a number of genes specific to mast cells. This mast cell TF network can take place in HSCs to regulate specific “primed” genes through the common HSC-mast CREs to regulate the expression of genes common to HSCs and mast cells.

Today, several hundred TF ChIP-Seq studies are available for a variety of hematopoietic cell types through the ENCODE project (80). This approach validates binding of TF proteins to CRE regions genome wide, making this strategy more convenient to build TF networks. It helps to improve our understanding of TF networks in the HSC context and validates networks previously predicted using the CRE strategy. For example, statistical analysis of 10 TFs-ChIP-seq data (TAL1, LYL1, LMO2, GATA2, RUNX1, MEIS1, PU.1, ERG, FLI-1, and GFI1B) performed in the multipotent mouse hematopoietic progenitor cell line 7 (HPC-7) has revealed a novel TF network consisting essentially of 7 TFs or the ‘heptad factors’ (TAL1, LYL1, GATA2, LMO2, ERG, FLI1, and RUNX1) that play important roles in regulating more than 900 genes expressed specifically in HSCs. Protein immunoprecipitation experiments in conjunction with mouse models (Gata2^+/-^ Runx1^+/-^ mice) have confirmed the central role of the interaction between GATA2 and RUNX1 and their dosage in maintaining the transcriptional activity of this TF network in HSCs and during the establishment of adult hematopoiesis (81). Interestingly, using the ChIP-seq strategy, this network of 7 TFs was shown to integrate with other TFs such as MITF and c-FOS to regulate the expression of genes specific to the mast cell context. Moreover, this mast cell TF network is also detected in HSCs where it presumably acts to regulate specific “primed” genes that are common to HSCs and mast cells (**Figure 4**)(82). The same phenomenon is observed in erythroid cells, where GATA1 can progressively replace GATA2 in the HSC TF network to regulate a specific set of genes in erythroid progeny (81, 83).

Whilst the application of the TF-ChIP-seq strategy in primary adult HSCs remains difficult due to the large amounts of starting material required, several methods that require fewer cells have been recently developed. For example, multiplexed, indexed T7 ChIP-seq (84) can be applied to a sample with fewer cells (85). Other studies have shown that ChIP-seq or related approaches such as CUT&Tag can be performed at the single cell level (84, 86, 87). Taken together, these technological advances are expected to greatly improve our understanding of TF networks in adult HSCs.

### 6.3 Application of TF networks for HSCs derivation strategies

Ground-breaking progress has been made in the field of allogeneic hematopoietic stem cell transplantation (HSCT), which remains the only curative treatment for many malignant and non-malignant diseases and the most widely used cellular therapy. It has also paved the way for the concepts of gene therapy and immunotherapy as tools to combat diseases that currently have no cure (88). Despite the rapid progress in the application of HSCT in the clinic, there are still some difficulties related to the availability of graft donors and their allogenicity. To compensate for the lack of a compatible donor optimally matched to the human leukocyte antigen (HLA), umbilical cord blood (UCB) has been used as an alternative. However, several studies have shown a lack of sufficient doses of stem and progenitor cells (HSPCs) to ensure long-term reconstitution of functional peripheral blood cells (89). Even after expansion of human HSPCs from UCB (CD34^+^ cells) using a clinically relevant Notch-mediated *ex vivo* expansion system, long-term transplantation of expanded HSPCs into patients remains limited (90).

Many efforts are being made to develop an efficient method to generate sufficient numbers of adult HSCs for research and therapeutic applications. The recent important discovery of somatic cell reprogramming by using TFs to induce pluripotent stem cells (iPSCs) (91) has strongly supported the applicability of TFs as an efficient strategy to generate functional adult HSCs. For example, the study of 16 TFs during the differentiation of ESCs into mature blood cells (e.g., macrophages) confirms that some “major” TFs are required throughout the differentiation process. Ectopic expression of these TFs (GATA2, LMO2, FLI1, and TAL1) in fibroblasts (nonhematopoietic cells) has been sufficient to generate mature blood cells *in vitro*, confirming their “instructive” role in overcoming phenotypic and epigenetic barriers imposed by normal developmental ontogeny (92).

The main goal of several studies is to improve the generation of functional HSCs that have long-term self-renewal capacity and the potential to differentiate into multiple cell lineages. The TF reprogramming strategy has been widely used to transform various cell types (terminally differentiated somatic cells, pluripotent cells, and hematopoietic progenitor cells) into functional HSCs. Somatic cells can be considered as the optimal cell type for generating HSCs due to their availability. For example, when MEFs (mouse embryonic fibroblasts) expressing the CD34 reporter mechanism (surface marker for hematopoietic cells) are used to screen 18 TFs, only a few TFs such as GATA2, ETV6, and GFI1B are able to reactivate the expression of the CD34 reporter system and promote the generation of myeloid colony-forming cells (93). In another study, ectopic expression of 6 TFs (including GATA2 and RUNX1c) was also shown to be able to generate hematopoietic cells from MEFs and adult fibroblasts. Despite the ability of derived hematopoietic cells to express a mixture of primitive and definitive globins and give rise to mature T lymphoid cells, their ability to transplant and establish long-term hematopoiesis *in vivo* remains limited (94).

The TF reprogramming strategy has also been applied to pluripotent stem cells (PSCs) and embryonic stem cells (ESCs) to generate hematopoietic cells. Indeed, screening of 27 candidate TFs revealed the existence of two TF networks capable of inducing distinct hematopoietic programs from human PSCs: pan-myeloid (ETV2 and GATA2) and erythro-megakaryocytic (GATA2 and TAL1). Interestingly, these two transcriptional networks directly convert human PSCs (hPSCs) into endothelial cells, which subsequently transform into hematopoietic cells with a tendency to myeloid or erythro-megakaryocytic potential. Furthermore, it shows that hematopoietic differentiation from hPSCs using these two distinct sets of hematopoietic TFs proceeds through the EHT stage (95). Other groups have directly used the highly purified nonhemogenic human umbilical vein endothelial cells or adult dermal microvascular endothelial cells to derive functional HSCs. Indeed, ectopic expression of TFs (such as FOSB, GFI1, RUNX1, and SPI1 TFs) in these cells in combination with the serum-free instructive vascular niche monolayer culture system can induce the growth of hematopoietic colonies containing cells with functional and immunophenotypic characteristics of hematopoietic stem cells (like-HSCs). These endothelial cells reprogrammed into human like-HSCs have the potential to transplant into immunodeficient mice after primary and secondary transplantation, with long-term differentiation into multiple lineages (96).

Thus, one might conclude that TF-mediated reprogramming of committed cells to hematopoietic lineages could increase the efficiency of the conversion process, since the distance in the developmental ontogeny of these cells from HSCs is relatively small compared to nonhematopoietic cells (e.g., fibroblasts). Indeed, ectopic expression of 6 TFs (RUN1T1, HLF, LMO2, PRDM5, PBX1, and ZFP37 TFs) is sufficient to reprogram adult mouse pre-Pro B cells and myeloid progenitors into induced HSCs (iHSCs) that have clonal differentiation potential for multiple cell lineages, reconstitute stem/progenitor compartments, and are serially transplantable. Moreover, single-cell gene expression analysis reveals the high similarity of iHSCs to adult HSCs. However, the stage of final hematopoiesis reached by this strategy is not yet clear. It appears that this conversion process occurs entirely within the confines of definitive hematopoiesis, without passing through the EHT stage (97).

Taken together, these studies illustrate the feasibility and applicability of TF-mediated conversion or directed differentiation strategies for the derivation of transplantable human hematopoietic cells from a variety of cell sources. A variety of TFs has been tested, highlighting the power of the TF network strategy for cell reprogramming and hematopoietic cell derivation. However, the therapeutic value of “derived HSCs” is still subject to certain limitations.

### 6.4 The lesson we can learn from malignant cells for better cell reprogramming of adult HSCs

The evolutionary process of leukemogenesis depends primarily on the origin of the malignant cells. Certain types of leukemia (e.g., AML) begin earlier in the hematopoietic hierarchy. Accumulation of mutations in HSCs during the lifespan results in preleukemic HSCs, which later develop into leukemic stem cells (LSCs)(98, 99). However, in other types of leukemia (e.g., B-cell lymphomas)(100), their origin may be in mature, post-mitotic, or non-proliferative cells, but the “disruptive” mutations may cause constitutive dysregulation of the regulatory networks responsible for cell lineage specification/differentiation, and this could enhance their plasticity so that they acquire some stem cell properties and become more “stem cell-like”. For example, the transcription factor PAX5 is known to be a master regulator of B lymphopoiesis (101), and its expression is required for the irreversible specification of HSC/immature progenitor cells to the B cell lineage (102). Indeed, heterozygous deletions of PAX5 are found in approximately one third of B-ALL patients (103), and the mouse model shows that the absence of PAX5 blocks the transition between pre-Pro-B and Pro-B stages during B differentiation. However, these Pax5^-/-^ B cell progenitors can trans-differentiate *in vitro* into different hematopoietic cell types (e.g., Macrophages, Granulocytes and naturel killer cells) (104) in the presence of appropriate cytokines and give rise to T lymphocytes *in vivo* after transplantation into RAG2-deficient mice (105) showing that they have acquired “stem-cell like” properties. It is also important to highlight the role that chromatin compaction plays in maintaining cell identity. In B-cell lymphomas, approximately 40% of patients have histone H1 mutations (HIST1H1B-E)(106). Histone H1 proteins act as a linker that binds to nucleosomes facilitating chromatin compaction (107). Their deletion (*H1c* and *H1e* alleles) in mice confers enhanced fitness and self-renewal properties to mature germinal center B cells, ultimately leading to aggressive lymphomas with increased repopulation potential. Interestingly, disruption of H1 function leads to a profound remodeling of genome architecture, causing a shift in chromatin from a compacted to a relaxed state in which genes related to stem cell functionality are upregulated (e.g., Klf4, Klf5, Meis1, Prdm5, Mycn, Spry2, and Hoxa9). Thus, alteration of H1 expression could increase cell plasticity by relaxing compact chromatin in mature cells, making them potential targets for TFs and facilitating cell reprogramming. Indeed, OSKM expression in H1c^-/-^; H1e^-/-^ murine embryonic fibroblasts resulted in a three- to fourfold increase in the efficiency of forming H1c/e-deficient iPSC colonies (106). Also, this cell plasticity of leukemic cells has been exploited to develop new therapeutic opportunities by trans-differentiation (reprogramming) of leukemic cells into non-malignant cells. In certain cases, primary human BCR-ABL^+^ B-ALL cells could be induced to reprogram into macrophage-like cells by exposing them to myeloid differentiation-promoting cytokines *in vitro* or by transiently expressing the myeloid transcription factor C/EBP alpha or PU.1. Interestingly, this myeloid reprogramming of B-ALL blasts abolishes their leukemogenicity *in vitro* and *in vivo* (108).

In summary, the transition of cells from one state to another (e.g., from stem cell to mature cell) is well regulated by multiple molecular networks. Several transcriptional and epigenetic factors play a role in maintaining cell identity, and their disruption may increase cell plasticity, which could be very useful for the reprogramming of adult HSCs. Recent advances in drug development targeting proteins such as PROTACs (Proteolysis Targeting Chimaera) may be useful for transient disruption of those proteins (e.g., TFs, linker histones, and epigenetic factors) to increase the cell plasticity and to facilitate the reprogramming of adult HSCs from any cell type.

## 7 Conclusion and perspective

It is estimated that billions of hematopoietic cells are produced daily in the human body (109). The hematopoietic stem cells (HSCs) compartment is composed of a small pool of cells capable of generating all blood cells and ensuring the durability of hematopoietic tissue throughout life. Its homeostasis is regulated by a complex set of external signals and intrinsic fate determinants. Among those, transcription factors (TFs) have been shown to play a central role in regulating hematopoiesis, particularly by influencing the self-renewal and differentiation of HSCs through orchestrating the coordinated expression of target genes as lineage establishment begins and progresses (110).

**Figure 5:**
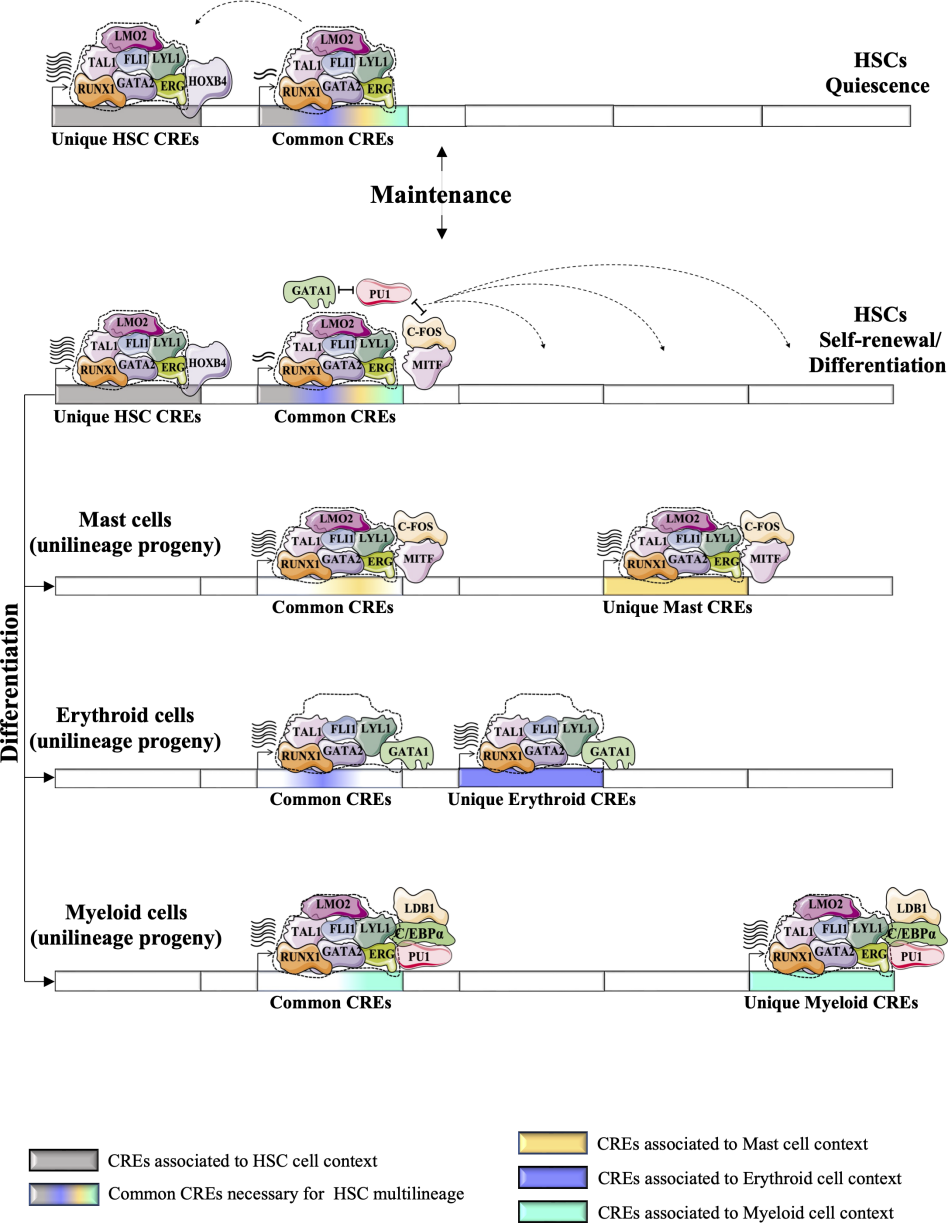
Proposed a model for the dynamics of HSC TF networks (e.g., heptad factors) during the resting state and during the cycle for self-renewal or/and differentiation. HSC TF networks could recognize unique CRE regions that regulate the expression of genes involved in self-identity/renewal of HSCs. It also recognizes the common CRE regions (or “primed” CREs), the key CREs for multilineage differentiation of HSCs, which are shared with different unilineage progeny and control the multilineage potential of HSCs. The unique HSC CREs could be more active during the resting state of HSCs, but during the cycle state, the HSC TF networks could promote the activity of the common CREs more strongly. Antagonism between TFs associated with different unilineage progeny (e.g. PU1, GATA1, MITF) positively affects the expression of genes associated with the common CREs. Different physiological conditions could promote the differentiation of HSCs into different progenitor cells. HSC TF networks could undergo several changes during the differentiation process by integrating new TFs or replacing HSC-expressed TFs with other TFs associated with the context of each lineage. These modifications enable the new formed TF networks to precisely recognize other CREs. The recycling of HSC TFs into alternative CREs is indicated by dashed arrows. Following the multilineage differentiation of HSCs (represented by bold arrows), the lineage-specific TF networks are capable of regulating gene expression through common CREs, as well as unique CREs that are specific to each particular unilineage context (such as mast, erythroid, and myeloid cells). (74).

Seminal studies have shown the importance of certain TFs for the quiescence of adult HSCs (e.g., GATA2 (28), GFI1/(35, 111)), others for their self-renewal (e.g., HOXB4 (112), EVI1 (113)) and differentiation (e.g., RUNX1 (114), TAL1(115)). These TFs may cooperate to form transcriptional networks that control the functions of HSCs and the emergence of adult hematopoiesis. This HSC TF network regulate a number of genes (encoding cell identity and self-renewal of HSCs) *via* CRE regions that are highly enriched for motif binding of TFs such as HOXB4, which is known to be involved in self-renewal of HSCs (82, 116). During the differentiation of HSCs into unilineage progeny, the HSC TF network undergoes some changes due to the integration of new TFs or the progressive replacement of TFs expressed by HSCs with other TFs associated with the unilineage context. These new lineage-specific TF networks could be initiated at the level of HSCs to regulate the expression of “primed” genes (117) that are highly active in the progeny of each lineage (e.g. erythroid, myeloid, mast) (**Figure 5**). The stochastic transcription of genes at the level of HSCs could be explained by the antagonistic effect between these less active lineage-specific TF networks, which indirectly reduces the transcription of “primed” genes and keeps HSCs in an “undifferentiated” state with multi-lineage potential (118). For example, PU.1 (SPI1), which is required for terminal myeloid differentiation, and GATA1, which plays a crucial role in erythroid differentiation (119), could both initiate different lineage-specific TF networks in HSCs, but the antagonism between PU.1 and GATA1 could prevent HSCs from differentiating into both erythroid and granulocyte/monocyte lineages (120). However, this balance between minor lineage-specific TF networks in HSCs can be disrupted by physiological conditions (e.g., acute stress and injury). For example, increasing the expression of GATA1 by the cytokine EPO in LT-HSCs enhances their differentiation toward the erythroid lineage (121). Absolute quantification of TFs by mass spectrometry in HSPCs has confirmed coexistence of the TF network of HSCs with other lineage-specific TF networks in the undifferentiated state (122). Additional studies are needed to further explore the dynamics of all TF networks present in adult HSCs. Recent advances in mass spectrometry technology (e.g., SWATH mass spectrometry (123)) could help to accurately assess the temporal dynamics of TF networks/complexes during the different states of HSCs (quiescence/cycling, self-renewal/differentiation)(124, 125) and to map their evolutionary hierarchy during the differentiation into different unilineage progeny.

The study of TF networks in adult HSCs has laid the groundwork for the development of several strategies to obtain transplantable hematopoietic cells for research and clinical applications. Several sets of defined TFs have been tested for their efficiency in reprogramming or directly differentiating adult HSCs from a variety of cell sources. However, the potential therapeutic value of these cells remains unknown, as the developmental state of the derived HSCs is incompletely characterized.

The challenge of generating functional human HSCs *ex vivo* for therapeutic purposes requires a better understanding of normal HSC biology through the use of multidisciplinary strategies. New technologies such as CRISPR/Cas could be used for high-throughput genetic screening to identify additional TFs that improve the efficiency of HSCs derivation or direct differentiation and to optimize their developmental state. This technology could also be used to test the efficiency of the different TFs previously used to derive transplantable hematopoietic cells side by side.

One of the major challenges in TF reprogramming of adult HSCs is the multilineage capability of the derived cells. The studies described above suggest that the multi-lineage capability of adult HSCs is provided by “primed” CRE regions that can be bound by different lineage-specific TF networks to activate transcription of primed genes that promote differentiation of HSCs into different lineages. These “primed” CRE regions are common to different unilineage cells, and their presence in adult HSCs is likely required for their multilineage potential. Also, limiting the activation of “lineage-specific” CREs during the process of reprogramming could be another avenue to limit the differentiation of *ex vivo* derived HSCs and promote their accumulation in the undifferentiated state. Recent studies of ZEB1/2 in adult HSCs have confirmed that those TFs play a role in repressing genes that are expressed only in differentiated cells at the HSC level (e.g., CSF1R, CD74 expressed in differentiated myeloid and lymphoid cells, respectively), underscoring the importance of “lineage-specific” CRE repression for lineage fidelity and the multilineage potential of HSCs (126). In addition, absolute protein quantification of TFs in HSPCs indicates that TFs co-repressors are significantly more abundant than co-activators (55, 122), which needs to be taken into account for the different TF cocktails used to reprogram HSCs, respecting the ratio of TFs activators to repressors that exists in HSC context, especially the presence of TFs involved in the repression of “primed” and “lineage-specific” CREs to limit the differentiation of derived HSCs and to maintain their potential (127).

The use of high-throughput genetic screening would be interesting to confirm this hypothesis and to uncover the molecular mechanisms involved in the activation or reprogramming of these common “primed” CRE regions in adult HSCs. These findings will help to improve the quality and potential of derived adult HSCs. Finally, screening of small molecules may provide more economical alternatives to the use of cytokines (e.g., UM171, SR1)(128, 129) and, in combination with all of the above, may help to develop a successful protocol for the derivation and expansion of functional adult HSCs that is suitable for clinical applications in hematological malignancies and beyond.

## Author contributions

AB drew up the concept of the review, wrote text, collected references, and made the first version of the Figures. JH wrote text and revised the Figures. MB drew up the concept of the review, wrote text, and revised the Figures. All the authors contributed to the article and approved the submitted version.
